# Giant tunnelling electroresistance in metal/ferroelectric/semiconductor tunnel junctions by engineering the Schottky barrier

**DOI:** 10.1038/ncomms15217

**Published:** 2017-05-17

**Authors:** Zhongnan Xi, Jieji Ruan, Chen Li, Chunyan Zheng, Zheng Wen, Jiyan Dai, Aidong Li, Di Wu

**Affiliations:** 1 College of Physics, Qingdao University, Qingdao 266071, China; 2 National Laboratory of Solid State Microstructures, Department of Materials Science and Engineering, and Collaborative Innovation Center for Advanced Materials, Nanjing University, Nanjing 210093, China; 3 Department of Applied Physics, The Hong Kong Polytechnic University, Hong Kong SAR 999077, China

## Abstract

Recently, ferroelectric tunnel junctions have attracted much attention due to their potential applications in non-destructive readout non-volatile memories. Using a semiconductor electrode has been proven effective to enhance the tunnelling electroresistance in ferroelectric tunnel junctions. Here we report a systematic investigation on electroresistance of Pt/BaTiO_3_/Nb:SrTiO_3_ metal/ferroelectric/semiconductor tunnel junctions by engineering the Schottky barrier on Nb:SrTiO_3_ surface via varying BaTiO_3_ thickness and Nb doping concentration. The optimum ON/OFF ratio as great as 6.0 × 10^6^, comparable to that of commercial Flash memories, is achieved in a device with 0.1 wt% Nb concentration and a 4-unit-cell-thick BaTiO_3_ barrier. With this thinnest BaTiO_3_ barrier, which shows a negligible resistance to the tunnelling current but is still ferroelectric, the device is reduced to a polarization-modulated metal/semiconductor Schottky junction that exhibits a more efficient control on the tunnelling resistance to produce the giant electroresistance observed. These results may facilitate the design of high performance non-volatile resistive memories.

## Abstract (web summary)

Alternative non-volatile memory designs are needed as the scaling of flash-based memories reaches its physical limits. By careful engineering, Xi *et al*. achieve ON/OFF ratios as great as 6.0 × 10^6^ in ferroelectric tunnel junction devices making them comparable to commercial flash memories.

The electrically switchable spontaneous polarization makes ferroelectric thin films promising candidate materials for non-volatile memories^1^,^2^. Ferroelectric random access memories have already been commercially available, which have the advantages of fast write speed and low power consumption^1^,^2^. However, the ferroelectric random access memories are based on capacitor structures and the readout is destructive, preventing the miniaturization of the devices, increasing the readout time and limiting the device lifetime^1^,^2^.

Recently, ferroelectric polarization switching has been achieved in ultrathin films only a few unit cells (u.c.) in thickness^3^,^4^,^5^,^6^,^7^. Resistive-type non-volatile ferroelectric memories, enabling high density integration and non-destructive readout, have been realized based on ferroelectric tunnel junctions (FTJs) that are composed of two metal electrodes separated by an ultrathin ferroelectric as the barrier^8^,^9^,^10^,^11^,^12^,^13^,^14^,^15^. The overall effective barrier of an FTJ can be modulated by the polarization reversal in the ferroelectric barrier, giving rise to non-volatile switching of the tunnelling resistance between a high (OFF state) and a low (ON state) value, that is, the so-called tunnelling electroresistance (TER)^8^,^9^,^10^. The TER characteristic has been demonstrated in BaTiO_3_ (BTO) ultrathin films using metallic (La_0.67_Sr_0.33_)MnO_3_ or SrRuO_3_ as one electrode and a conductive tip in a scanning probe microscope as the other electrode^11^,^12^. Later, Chanthbouala *et al*.^13^ reported a high speed write/read with nanosecond pulses and a power consumption as low as femto-joule per bit in Au/Co/BTO/(La_0.67_Sr_0.33_)MnO_3_ FTJs. The TER behaviour has also been observed in a number of other metal/ferroelectric/metal tunnel junctions, such as Co/Pb(Zr,Ti)O_3_/(La_0.70_Sr_0.30_)MnO_3_ (ref. 14), Pt/BTO/SrRuO_3_ (ref. 16), Cr/BTO/Pt (ref. 17), (La_0.67_Sr_0.33_)MnO_3_/BiFeO_3_/(La_0.67_Sr_0.33_)MnO_3_ (ref. 18) and Co/BiFeO_3_/LaNiO_3_ (ref. 19), in which the ON/OFF current ratio originates from a modulation on the height of the ferroelectric barrier. Recently, efforts have been made to enhance the TER performance by incorporating extra barriers between the ferroelectric barrier and the metallic electrode, for example, the CoO_x_ layer at Co/BTO interface^20^, the ultrathin dielectric SrTiO_3_ barrier at BTO/SrRuO_3_ interface^21^,^22^ and the metal-insulator phase transition insertion (La_0.50_Ca_0.50_)MnO_3_ at BTO/(La_0.70_Sr_0.30_)MnO_3_ interface^23^. In these FTJs, the extra barriers vary in height and/or in width in response to the polarization reversal and give a more efficient modulation on the junction transport.

Previously, we have proposed a metal/ferroelectric/semiconductor (MFS)-type FTJ. In this device, there is an extra Schottky barrier on the depleted semiconductor surface, which varies both in width and in height in response to the ferroelectric polarization reversal due to a ferroelectric field effect. The non-volatile modulation on both the ferroelectric barrier and the extra Schottky barrier gives rise to a greatly enhanced ON/OFF ratio^24^. Using Nb:SrTiO_3_ (NbSTO) as the semiconductor electrode, an ON/OFF ratio of 10^4^ has been achieved in a Pt/BTO/NbSTO device^24^. More recently, Hu *et al*.^25^ have reported a variation of electroresistance by about ten times in ITO/Pt/BiFeO_3_/NbSTO FTJs under UV illumination, due to an optical modulation on the Schottky barrier in NbSTO. These results demonstrate the significance of the interface Schottky barrier on the performance of MFS tunnel junctions.

Here we show that, by engineering the Schottky barrier, a giant ON/OFF ratio, about 6.0 × 10^6^, can be achieved in an optimized Pt/BTO/NbSTO FTJ with a 4 u.c.-thick BTO barrier and a Nb concentration of 0.1 wt%. In particular, the 4 u.c.-thick BTO barrier provides a switchable polarization but shows a negligible resistance to electron tunnelling, as compared with the contribution from the Schottky barrier. Therefore, the TER comes completely from a polarization-modulation on the metal/semiconductor interface Schottky barrier. This is different from previously reported MFS-type FTJs^24^, where the interface Schottky barrier works together with the ferroelectric barrier. This polarization-modulation produces a more efficient control on the tunnelling transport through the device and generates the giant ON/OFF ratio comparable to commercial Flash memories^26^,^27^,^28^,^29^.

## Results

### Ferroelectric properties of ultrathin BTO films

**Figure 1:**
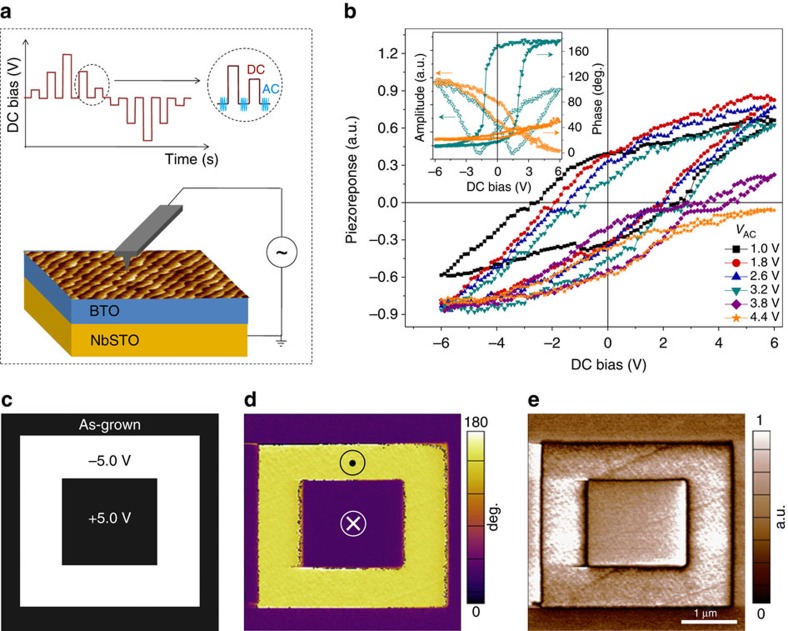
Topography and ferroelectric properties of a 4 u.c.-thick BaTiO_3_ film deposited on Nb:SrTiO_3_ substrate. (**a**) Atomic force microscopy surface morphology and a schematic description of the piezoresponse force microscopy (PFM) measurements. The inset shows the applied pulse train for hysteresis measurements. (**b**) Remnant PFM piezoresponse hysteresis loops collected with various *V*_AC_ values. The inset shows amplitude and phase hysteresis loops with *V*_AC_=3.2 V (dark green) and 4.4 V (orange). (**c**) Protocol for domain patterning. (**d**) PFM phase and (**e**) PFM amplitude images acquired after the domain patterning.

Piezoresponse force microscopy (PFM) is used to characterize ferroelectric properties of the ultrathin BTO films. As shown in Fig. 1a, the PFM signal is collected with a conductive tip in contact with the film surface, as the NbSTO electrode is grounded. The step-terrace surface morphology with a step height of 0.4 nm, measured by atomic force microscopy (AFM), indicates a layer-by-layer growth, which is also demonstrated by the intensity oscillation in reflection high energy electron diffraction collected *in situ* during the deposition (Supplementary Fig. 1). PFM hysteresis loops are measured from bare BTO surface using the waveform shown schematically in the inset in Fig. 1a, where a pulse sequence following a triangle profile is used to switch the film and the remnant piezoresponse is measured by a small-signal AC voltage (*V*_AC_) applied following each pulse^30^. The remnant piezoresponse hysteresis loop (see Methods section) of a 4 u.c.-thick BTO measured with *V*_AC_=1.0 V is shown in Fig. 1b. However, the PFM measurement is sensitive to the contact between the tip and the sample surface and similar hysteresis loops have also been observed in non-ferroelectric materials, such as LaAlO_3_/SrTiO_3_ heterostructures and amorphous HfO_2_ thin films, owing to charge injection, ionic motion and interfacial electrochemical reaction^31^,^32^. To distinguish the piezoresponse hysteresis due to ferroelectric polarization reversal, Strelcov *et al*.^33^ proposed a *V*_AC_-dependent PFM measurement, in which the remnant piezoresponse hysteresis loop of a ferroelectric material deforms and even collapses when *V*_AC_ is above the coercive voltage because the polarization flipping during measurement cancels the piezoresponse. However, this reduction of the measured piezoresponse above a critical *V*_AC_ value is absent in non-ferroelectrics^31^,^32^. The piezoresponse loops as a function of *V*_AC_ are shown in Fig. 1b. The hysteresis starts to reduce at *V*_AC_=3.2 V, just above the coercive voltage, and then reduces abruptly as *V*_AC_ further increases, in good agreement with the results reported previously in Pb(Zr,Ti)O_3_ and BiFeO_3_ thin films^31^,^32^. The PFM amplitude and phase loops measured at *V*_AC_=3.2 and 4.4 V are shown in the inset for clarity. Similar reduction in piezoresponse when *V*_AC_ is greater than the coercive voltage is also observed from Pt top electrode deposited on the BTO/NbSTO heterostructure (Supplementary Fig. 2a–c). These observations evidence robust ferroelectricity of the 4 u.c.-thick BTO film deposited on NbSTO. Figure 1d,e shows PFM out-of-plane phase and amplitude images of domains on this BTO film patterned according to the protocol shown in Fig. 1c. The 180° phase contrast and the clear domain boundary indicate that antiparallel domains can be written in this ultrathin BTO layer.

### The effects of ferroelectric barrier thickness

**Figure 2:**
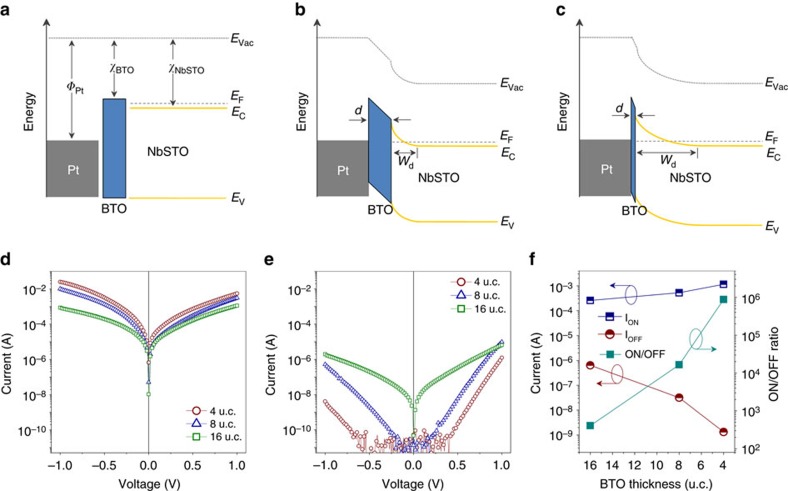
Tunnelling electroresistance of Pt/BaTiO_3_ (BTO)/Nb:SrTiO_3_ (NbSTO) ferroelectric tunnel junctions with various BTO thickness. Band diagram of separated Pt, BTO and NbSTO (**a**) and of Pt/BTO/NbSTO junction with a thick (for example, 16 u.c.) (**b**) and a thin (for example, 4 u.c.) BTO barrier (**c**), where *Φ*_Pt_=5.65 eV is the work function of Pt, *χ*_BTO_=3.9 eV the electron affinity of BTO, *χ*_NbSTO_=4.08 eV the electron affinity of NbSTO, *E*_Vac_ the vacuum level, *E*_C_, *E*_V_ and *E*_F_ the conduction band minimum, the valence band maximum and the Fermi level of NbSTO, respectively, *d* the BTO thickness, *W*_d_ the depletion region width. (**d**,**e**) *I*–*V* curves of Pt/BTO/NbSTO (Nb: 0.7 wt%) FTJs, with a BTO thickness of 4, 8 and 16 u.c., in the ON and the OFF states, respectively. (**f**) Junction currents and corresponding ON/OFF ratios, read at 0.6 V, as a function of BTO thickness.

The FTJ devices are defined by depositing Pt top electrodes on BTO/NbSTO heterostructures. Figure 2a depicts band alignment of separated Pt, BTO and NbSTO, assuming for clarity that the BTO is not polarized. The contact potential, arising from the difference between the work function of Pt (*Φ*_Pt_=5.65 eV) and the Fermi level (*E*_F_) of NbSTO, is shared by the BTO barrier layer and the depleted region on the surface of NbSTO (Fig. 2b)^34^,^35^. A Schottky barrier appears even though the BTO is assumed unpolarized. With decreasing BTO thickness, the potential that drops on the BTO layer decreases and the Schottky barrier on the depleted semiconductor surface enhances accordingly, as shown in Fig. 2c. The evolution of the energy profile with decreasing BTO thickness is reminiscent of the formation of a metal/semiconductor Schottky junction^34^. If the BTO thickness decreases to zero, the Pt/BTO/NbSTO structure reduces to a Pt/NbSTO Schottky contact. When the BTO barrier with a finite thickness is polarized, the ferroelectric bound charges at the semiconductor surface modulate the profile of the Schottky barrier via a ferroelectric field effect. This modulation results in the polarization-dependent transport through the junction, that is, the TER effect. This TER behaviour is confirmed by combined PFM and conductive AFM measurements on the Pt top electrode of a Pt/BTO/NbSTO device, as shown in Supplementary Fig. 2d–f. With the polarization pointing to NbSTO, the positive bound charges tend to suppress the Schottky barrier since the depleted region on the semiconductor surface is reduced or even annihilated by electron accumulation. The device is in the ON state with a larger current. As the polarization is switched pointing to Pt, the negative bound charges on the semiconductor surface help to sweep the electrons away from the ferroelectric/semiconductor interface and enhances the Schottky barrier. The junction current is suppressed associated with the appearance of a typical rectifying character. The device is then switched to the OFF state.

Room-temperature resistance-voltage hysteresis loops of the Pt/BTO/NbSTO (Nb: 0.7 wt%) FTJs as a function of BTO thickness are shown in Supplementary Fig. 3. The critical voltages for resistance switching between the ON and the OFF states coincide with the coercive voltages of the PFM hysteresis loops, which again evidences the correlation between resistive switching and the polarization reversal. Room-temperature current–voltage (*I*–*V*) curves for the ON and the OFF states of the Pt/BTO/NbSTO (Nb: 0.7 wt%) FTJs as a function of BTO thickness are shown in Fig. 2d,e, respectively. The write pulse height was determined from the resistance-voltage hysteresis loop for each device (Supplementary Fig. 3). Compared with the ON state transport characteristics in Fig. 2d, the OFF state *I*–*V* curves in Fig. 2e show a more pronounced variation with decreasing BTO thickness. Figure 2f shows the ON and the OFF current, as well as the ON/OFF ratio, as a function of BTO thickness. It is clear that the ON state current exhibits only a slight variation, while the OFF state current decreases about three orders of magnitude with decreasing BTO thickness from 16 down to 4 u.c., resulting in a dramatic increase in ON/OFF ratio from about 400 to 8 × 10^5^. The suppression of the OFF state current with decreasing BTO thickness is associated with the appearance of a rectifying transport character (Fig. 2e). This indicates that the Schottky barrier becomes more prominent in FTJs with a thinner BTO barrier, as shown in Fig. 2a–c. In fact, it is observed that the *I*–*V* characteristic of an In/BTO/NbSTO device with the thinnest BTO barrier is almost identical to an In/NbSTO Ohmic contact (Supplementary Fig. 4), indicating that the 4 u.c.-thick ultrathin BTO layer exhibits a negligible resistance to the tunnelling current. The Pt/BTO/NbSTO FTJ with a 4 u.c.-thick BTO barrier is then reduced to a polarization-modulated Pt/NbSTO metal/semiconductor Schottky junction. Figure 2f shows that such a modulation on the Schottky junction generates an improved ON/OFF ratio, as a result of the efficient shut-off of the OFF state current due to the enhanced Schottky barrier.

### The effects of Nb doping concentration

**Figure 3:**
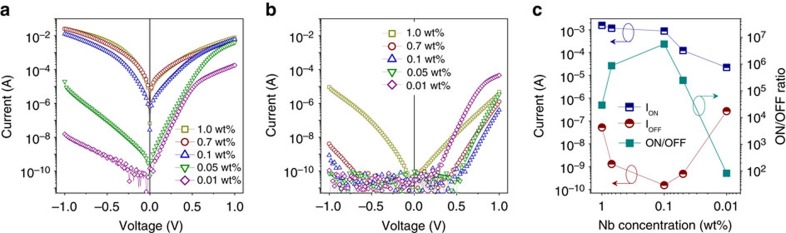
Tunnelling electroresistance of Pt/BaTiO_3_ (BTO)/Nb:SrTiO_3_ ferroelectric tunnel junctions with a 4 u.c.-thick BTO barrier as a function of Nb concentration. (**a**,**b**) *I*–*V* curves for the ON and the OFF state, respectively. (**c**) Junction currents and ON/OFF ratios, read at 0.6 V.

The TER characteristics of the polarization-modulated metal/semiconductor Schottky junction can be further improved by optimizing the Nb doping concentration in NbSTO. *I*–*V* curves of the ON and the OFF states for Pt/BTO/NbSTO FTJs with a 4 u.c.-thick BTO barrier and various Nb concentration from 0.01 to 1.0 wt% are shown in Fig. 3a,b, respectively. The ON state *I*–*V* curves at the Nb concentrations of 1.0 and 0.7 wt% are relatively symmetric for the forward and the reverse bias. However, it becomes more and more asymmetric with decreasing Nb concentration. The OFF state current shows a clear rectifying character, except for the junction with the highest Nb concentration of 1.0 wt%. The junction currents and the corresponding ON/OFF ratios are plotted in Fig. 3c, as a function of Nb concentration. As shown, the ON/OFF ratio increases from 3 × 10^4^ to 6 × 10^6^ with decreasing Nb concentration from 1.0 to 0.1 wt%, owing to the dramatic decrease of the OFF state current. The suppression of the OFF state current is accompanied with the increase in the onset voltage of the forward current (Fig. 3b). However, as the Nb concentration further decreases from 0.1 to 0.01 wt%, the ON/OFF ratio decreases abruptly. It reduces to only about 80 at the lowest Nb concentration of 0.01 wt%, as shown in Fig. 3c. With the decrease of TER, the ON state current decreases by more than an order (Fig. 3a), whereas the OFF state current increases significantly by almost four orders. In addition, the increase of OFF state current is associated with the decrease in the onset voltage of the forward current (Fig. 3b).

**Figure 4:**
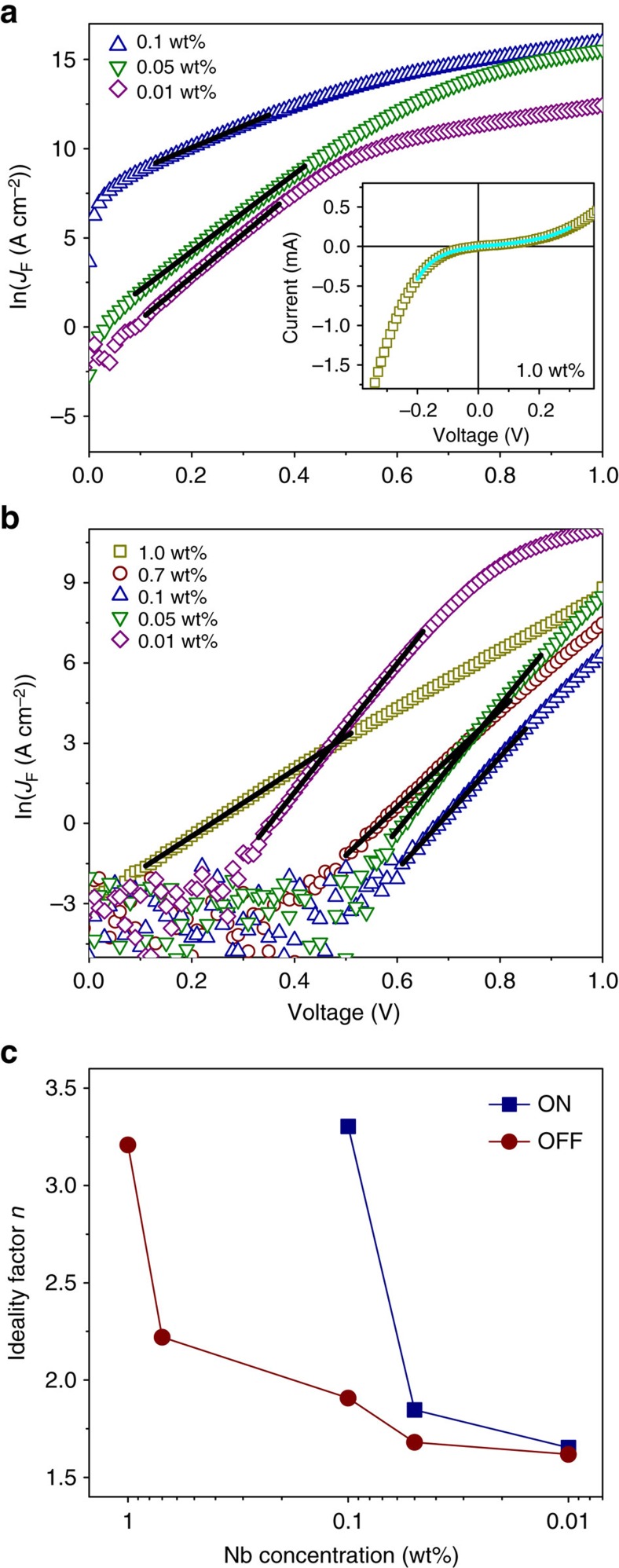
Fits to the *I*–*V* curves of Pt/BaTiO_3_ (BTO)/Nb:SrTiO_3_ ferroelectric tunnel junctions with a 4 u.c.-thick BTO barrier. (**a**,**b**) ln*J*_F_–*V* plots for the ON and the OFF states, respectively. The black solid lines are fits to equation (1). The inset in **a** is the ON state *I*–*V* curve of the junction with a Nb concentration of 1.0 wt%, where the cyan solid line is a fit to the direct tunnelling model. (**c**) Ideality factor *n* extracted from the fitting as a function of Nb concentration.

To gain insight into the complicated TER characteristics, the *I*–*V* curves are fitted to different transport models. In the heavily doped regime with Nb concentration at 1.0 and 0.7 wt%, the ON state *I*–*V* curves are identical and nonlinear. The low-voltage part of the *I*–*V* curves can be well fitted to the direct tunnelling model based on a trapezoidal potential barrier (see Methods section)^12^. As shown in the inset in Fig. 4a, the validity of the direct tunnelling model implies that the Schottky barrier on the heavily doped NbSTO surface is eliminated when the BTO polarization points to the NbSTO in the ON state. The BTO barrier, extracted from the fitting, is 1.7 and 0.2 eV in height for the Pt/BTO and the BTO/NbSTO interfaces, respectively. The *I*–*V* curve at higher voltage is dominated by Fowler–Nordheim tunnelling through a triangular barrier, as reported previously in metal/ferroelectric/metal FTJs^10^,^36^. As the Nb concentration decreases, the ON state currents at forward bias fit best to an exponential increase with the applied voltage at low voltage (Fig. 4a), suggesting the depletion of the NbSTO surface and the dominance of the Schottky barrier on the transport. However, the transport at higher voltage is dominated probably by a bulk-limited conduction mechanism^37^. On the other hand, as BTO polarization points to the Pt electrode in the OFF state, the Schottky barrier is enhanced due to the ferroelectric field effect and the *I*–*V* curves, in all the FTJ devices, exhibit a thermally activated character. The junction transport controlled by a Schottky barrier is given by^34^


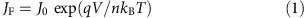


where *J*_F_ is the forward current density, *J*_0_ the saturated current density, *q* the electron charge, *n* the ideality factor, *k*_B_ the Boltzmann constant and *T* the absolute temperature. As reported previously, *n* equals unity if the transport is an ideal thermionic emission over a Schottky barrier^34^,^38^,^39^,^40^,^41^,^42^. *n* extracted from the fits is plotted in Fig. 4c as a function of Nb concentration. It is clear that *n* decreases as the device is switched from the ON to the OFF state and *n* decreases from 3.4 to 1.6 with decreasing Nb concentration from 1.0 to 0.01 wt%. The deviation of *n* from unity may be ascribed to thermally assisted tunnelling as observed previously in NbSTO-based Schottky junctions^34^,^38^,^39^,^40^ and the presence of the ultrathin BTO layer, which shares the voltage applied on the junction.

In Pt/BTO/NbSTO FTJs, *n* can be expressed as^39^,^40^,


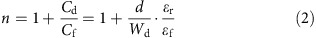


where *C*_d_ and *C*_f_ are the high-frequency capacitances of the depleted region and the BTO barrier layer, *ɛ*_r_ and *ɛ*_f_ their respective relative dielectric constants, and *W*_d_ the depleted region width. Here, *ɛ*_r_ of NbSTO is assumed to be 290 at room temperature, following Barrett's formula (Supplementary Information)^41^. According to equation (2), with a certain BTO thickness, the decrease of *n* indicates the increase in *W*_d_. The build-in potential (*V*_bi_) of a metal/semiconductor Schottky barrier is often analysed by capacitance-voltage measurements. However, in the present study with the presence of the ultrathin BTO layer, the depleted region capacitance *C*_d_ should be *nC*, where *C* is the high-frequency capacitance of the whole junction, and the voltage that drops on the depleted region (*V*_d_) should be calibrated as *V/n*, where *V* is the voltage applied.

**Figure 5:**
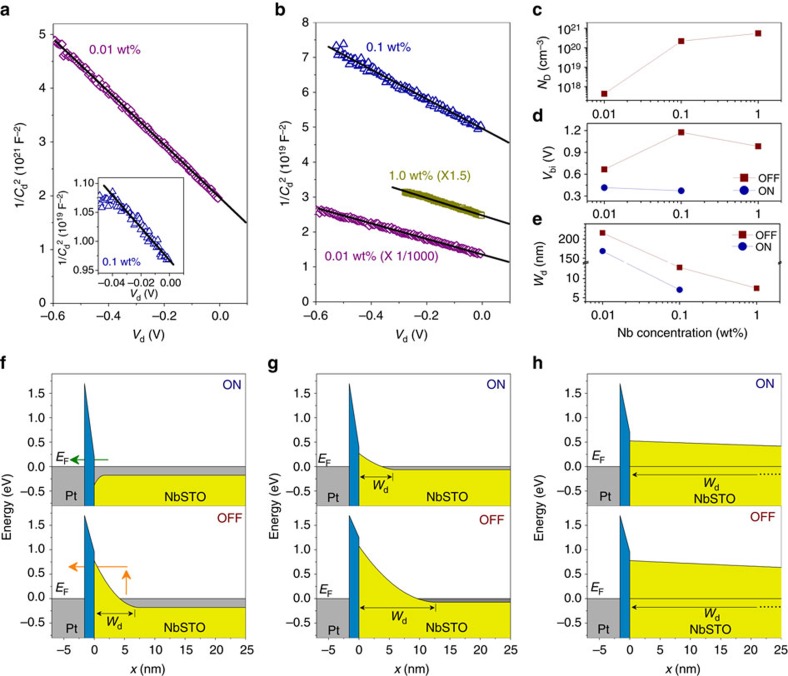
*C*–*V* characteristics and energy profiles of Pt/BaTiO_3_ (BTO)/Nb:SrTiO_3_ ferroelectric tunnel junctions with a 4 u.c.-thick BTO barrier and various Nb concentration. (**a**,**b**) 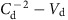 plots for the ON and the OFF states, respectively, where the black solid lines are fits to 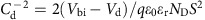. The inset in **a** is the ON state 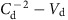 of the 0.1 wt% device. (**c**) *N*_D_ and (**d**) *V*_bi_, extracted from the fits, as a function of Nb concentration. (**e**) *W*_d_ of the ON and the OFF states as a function of Nb concentration. (**f**,**g**,**h**) Energy profiles at zero bias for the ON and the OFF states of the junctions with Nb concentrations of 1.0, 0.1 and 0.01 wt%, respectively, where the green and the orange arrows denote the direct tunnelling and the thermally assisted tunnelling, respectively.

The room-temperature 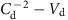 characteristics of the Pt/BTO/NbSTO FTJs with a 4 u.c.-thick BTO barrier for the ON and the OFF states are shown in Fig. 5a,b, respectively, as a function of Nb concentration. The ON state capacitance for the device with the Nb concentration at 1.0 wt% is not shown because the Schottky barrier is completely annihilated by the electron accumulation. As shown, 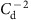 increases linearly with reverse bias, indicating that the permittivity of NbSTO is constant in the depleted region^35^,^42^,^43^. Similar linear 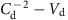 behaviours have also been observed in Au/NbSTO Schottky junctions, in which the *ɛ*_r_ of NbSTO exhibits a significant dependence upon electric field only at low temperature below 100 K (ref. 42). Therefore, the 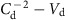 plots can be fitted using 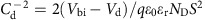, which yields the doping concentration *N*_D_ in NbSTO from the slope and the build-in potential *V*_bi_ of the Schottky barrier from the intercept. *N*_D_ values extracted are plotted in Fig. 5c, which are comparable to those reported previously in metal/NbSTO Schottky junctions^38^,^43^,^44^. *V*_bi_ at zero bias are shown in Fig. 5d. With *V*_bi_ and *N*_D_, *W*_d_ can be estimated from *W*_d_=(2*ɛ*_0_*ɛ*_r_*V*_bi_/*qN*_D_)^1/2^, as shown in Fig. 5e. It is obvious that *V*_bi_ and *W*_d_ in the OFF state is larger than those in the ON state, indicative of the enhanced Schottky barrier in the OFF state and the effective ferroelectric modulation on it.

The energy profile of the Schottky barrier can be deduced in terms of *V*_bi_ and *W*_d_ obtained above for each Nb concentration (see Methods section), as shown in Fig. 5f–h. For heavily doped NbSTO with Nb concentration at 1.0 and 0.7 wt%, the Schottky barrier at the BTO/NbSTO interface is absent in the ON state. Therefore, the transport is governed by direct electron tunnelling through the 4 u.c.-thick BTO barrier (Fig. 5f), as observed in Fig. 3a. Thus, the large ON state current is observed. When the Nb concentration decreases to 0.1 wt%, the Schottky barrier appears. This leads to the thermally activated *I*–*V* characteristic observed in the ON state, as shown in Fig. 3c. The direct tunnelling may be suppressed because of the increase in the effective barrier width at *E*_F_ with the appearance of the Schottky barrier, which is 3.87 nm in width and 0.26 eV in height, as extracted from the fitting. However, the thermally assisted tunnelling, in which the electrons are thermally emitted to an energy above *E*_F_ and then tunnel through a thinner barrier, is still pronounced with this weak Schottky barrier. This gives the *n* value greater than unity, as observed in Fig. 4c. The existence of thermally assisted tunnelling currents is confirmed by further analysing temperature-dependent *I*–*V* curves (Supplementary Fig. 5)^25^,^42^,^45^. Therefore, the FTJ with the Nb concentration at 0.1 wt% exhibits a large ON state current comparable to the direct tunnelling through the BTO barrier (Fig. 3a). As the Nb concentration further decreases, both *V*_bi_ and *W*_d_ increase in the ON state. The device with the Nb concentration at 0.01 wt% exhibits a Schottky barrier of 0.40 eV in height and 168 nm in width. The thermally assisted tunnelling through the barrier is hence suppressed, in comparison with the transport in the 0.1 wt% device, and the ON state current decreases, as observed in Fig. 3a,c.

In the OFF state, the surface of heavily doped NbSTO, with the Nb concentration at 1.0 and 0.7 wt%, is switched to depletion. For the device with the Nb concentration at 1.0 wt%, the Schottky barrier is 0.76 eV in height but only 4.02 nm in width. This thin barrier cannot suppress the thermally assisted tunnelling completely. When the Nb concentration decreases to 0.1 wt%, the Schottky barrier increases to 1.07 eV in height and 9.68 nm in width. The OFF state current is then efficiently shut off as the barrier gets high enough and wide enough to suppress the thermally assisted tunnelling. Therefore, a greatly enhanced ON/OFF ratio is achieved at this composition (Fig. 3f). As the Nb concentration further decreases, *E*_F_ drops into the bandgap of NbSTO (see Methods section) and the contact potential is reduced. With Nb concentration at 0.01 wt%, *V*_bi_ is only 0.64 V, although the barrier extends deep into NbSTO (*W*_d_=216 nm), as shown in Fig. 5h. With this low Schottky barrier height, the thermally assisted tunnelling becomes pronounced again since the electrons are easier to be emitted to an energy level where the barrier is thin enough to allow the tunnelling. This is in agreement with the observation that the onset of the OFF state current decreases when Nb concentration decreases from 0.1 to 0.01 wt%. This larger OFF state current results in the smaller ON/OFF ratio observed in this lightly doped composition range, as shown in Fig. 3c.

**Figure 6:**
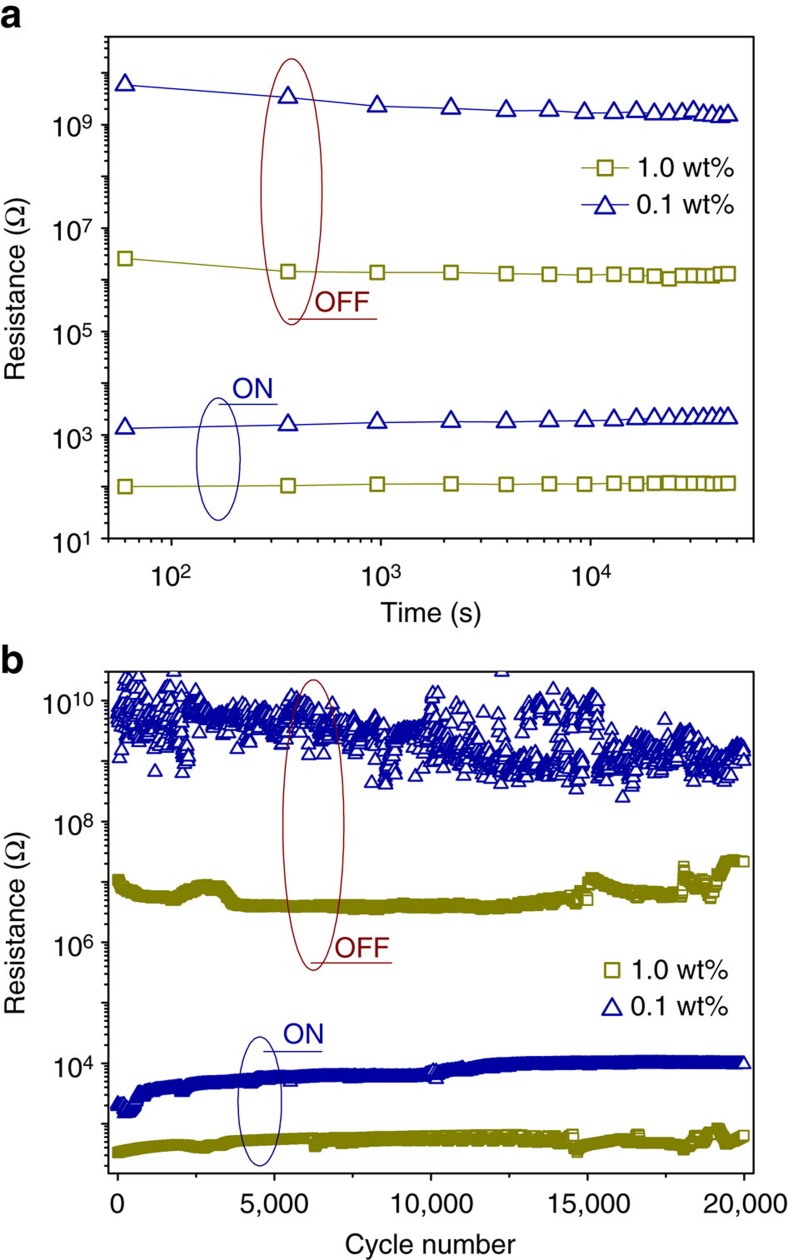
Room-temperature device endurance of the 4 u.c.-thick Pt/BaTiO_3_/Nb:SrTiO_3_ ferroelectric tunnel junctions with Nb concentrations of 1.0 and 0.1 wt%, respectively. (**a**) Data retention and (**b**) bipolar resistance switching. Cycling pulse voltages are +2.5/−3.0 V and +4.5/−5.0 V for the 1.0 and 0.1 wt% FTJs, respectively. The read pulse voltage is 0.6 V.

From the above analyses, one may find that in FTJs with a 4 u.c.-thick BTO barrier, the junction transport is predominantly controlled by the interface Schottky barrier and the BTO ferroelectric barrier itself is negligible to the transport because the ultrathin barrier allows a large tunnelling transmittance, as evidenced in Supplementary Fig. 4. The highest ON/OFF ratio observed at 0.1 wt% Nb concentration is a result of the optimized Schottky barrier, which efficiently modulates the thermally assisted tunnelling current between the two resistance states. The optimized FTJ with 0.1 wt% Nb concentration exhibits excellent resistance retention with no obvious degradation observed within 12 h in both the ON and the OFF states, as shown in Fig. 6a. After 2 × 10^4^ bipolar switching cycles, the device still maintains a giant ON/OFF ratio about 1.0 × 10^5^, as shown in Fig. 6b, indicating excellent switching endurance. Data retention and bipolar switching properties of the FTJ with 1.0 wt% Nb concentration are shown in Fig. 6 for comparison. In spite of the lower ON/OFF ratio, it also shows good retention and switching endurance.

## Discussion

We have shown that TER in Pt/BTO/NbSTO FTJs can be greatly enhanced by engineering the Schottky barrier. The ON/OFF ratio increases from about 400 to 8 × 10^5^ by simply decreasing the BTO thickness from 16 to 4 u.c., where the OFF state current is significantly suppressed due to the enhancement in the Schottky barrier. The TER is further optimized with varying Nb concentration. For FTJs with heavily doped NbSTO, the ON/OFF ratio is limited by the large OFF state current since the Schottky barrier is too thin to suppress the electron tunnelling. For FTJs with lightly doped NbSTO, the Schottky barrier height is low even though the barrier width is large. This low barrier height facilitates thermally assisted tunnelling in the OFF state and degrades the ON/OFF ratio. With an optimized Nb concentration at 0.1 wt%, the Schottky barrier is appropriate both in width and in height, which allows a large ON state current but shuts off the current efficiently in the OFF state, giving rise to the highest ON/OFF ratio about 6 × 10^6^.

Recently, Liu *et al*.^46^ have reported that, in addition to the Schottky barrier, the width of the ferroelectric barrier itself can also be modulated in SrRuO_3_/BTO/n-SrTiO_3_ FTJs, where the BTO surface is metallized in the ON state, due to penetrated electrons from the accumulated n-type SrTiO_3_ surface. This reversible metallization probably cannot be ruled out in the FTJs with heavily doped NbSTO. However, the largest ON/OFF ratio observed comes from the efficient modulation on thermally assisted tunnelling through a Schottky barrier with an appropriate profile. This highlights the effect of Schottky barrier engineering in performance optimization in MFS-type FTJs. The predominant function of the Schottky barrier is also evidenced by the drastically reduced TER (only about 10, as shown in Supplementary Fig. 6) observed in an Al/BTO/NbSTO (Nb: 0.1 wt%) FTJ with a 4 u.c.-thick BTO barrier, in which the Schottky barrier is significantly suppressed due to the low work function (about 4.28 eV) of the Al top electrode.

In summary, the proposed Schottky barrier engineering provides a convenient approach to optimize the TER performance by tuning microstructural parameters. We find that with a 4 u.c.-thick ultrathin BTO barrier, the MFS-type FTJs may be regarded as polarization-modulated metal/semiconductor Schottky junctions, in which an efficient control on the junction transport and a greatly enhanced TER are achieved. Chang and Esaki have proposed a non-volatile Schottky diode based on a MFS tunnelling structure, in which the Schottky barrier height can be modulated by charge/discharge of interfacial states associated with the polarization reversal^47^. In the present work, coherent growth of BTO on lattice-matched NbSTO may greatly suppress the interfacial states^24^,^48^ and the observed TER is from the modulation on the depleted space charge region and hence the Schottky barrier with the polarization reversal. Recently, high-quality HfO_2_-based ferroelectric ultrathin films have been elaborated directly on Si substrates^49^,^50^,^51^. With appropriate choice of material parameters, the proposed ferroelectric-modulated Schottky junctions may have potential applications for Si-based memories compatible to the current technology. In addition, the electrically tunable rectifying characteristics and the giant ON/OFF ratio in these devices may help to suppress crosstalk issues in two-terminal crossbar architectures and facilitate the design of high performance memory devices^52^,^53^.

## Methods

### Device preparation

Ultrathin BTO films with thickness of 4–16 u.c. were epitaxially grown on (001) single-crystalline NbSTO (Nb: 0.01–1.0 wt%) substrates by pulsed laser deposition using a KrF excimer laser (Coherent COMPexPro 201), monitored *in situ* by reflection high energy electron diffraction. Prior to the deposition, the NbSTO substrates were etched by NH_4_F buffered-HF solution and then annealed at 950 °C for 1 h in flowing O_2_ to form a TiO_2_ single-terminated step-terrace surface. The BTO films were deposited with 2.5 J cm^−2^ laser energy density at 2 Hz repetition, keeping the substrate temperature at 750 °C and the O_2_ pressure at 5 × 10^−3^ mbar. Pt top electrodes about 30 μm in diameter and 200 nm in thickness were deposited on the surface of BTO/NbSTO heterostructures by sputtering with a shadow mask to form the FTJs.

### Electrical characterizations

The surface morphologies of the BTO/NbSTO heterostructures were recorded by a Park XE-07 AFM. Room-temperature ferroelectric properties of the BTO/NbSTO heterostructures were measured using an Asylum Research Cypher scanning probe microscope with conductive Pt/Ti-coated tips as the top electrodes. The PFM hysteresis loops were collected in the DART (dual a.c. resonance tracking) mode with triangle pulse waveforms applying on the tip. The piezoresponse (*PR*) is deduced from the amplitude (*A*) and the phase (*θ*) signals by the formula of *PR=A*cos*θ*. Phase and amplitude images were recorded in the single-frequency PFM mode. Conductive AFM is measured on the Pt electrode of a Pt/BTO/NbSTO FTJ with bias applied on a doped diamond-coated tip (CDT-NCHR, NanoWorld). The resistance switching of the Pt/BTO/NbSTO FTJs were measured by a Keithley 2400 SourceMeter and the low temperature *I*–*V* measurements were performed on a LakeShore CRX-4K probe station. *C*–*V* characteristics were measured using an Agilent 4294A impedance analyser at room temperature with a frequency of 4 MHz and an oscillation level of 50 mV. The testing pulses were applied to the Pt electrodes and the NbSTO substrates were always grounded through indium ohmic contact pads.

### Direct tunnelling model

For a trapezoidal potential barrier, the current density *J* can be described by^12^


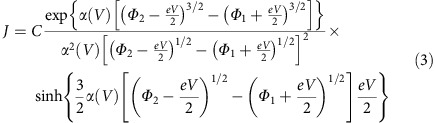


where 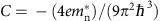 and 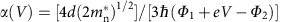, *Φ*_1_(*Φ*_2_) is the barrier height at the Pt/BTO (BTO/NbSTO) interface, 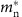 the effective electron mass, *ħ* the reduced Planck constant, and *d* the BTO barrier width (approx. 1.6 nm).

### Schottky barrier calculation

Taking *E*_C_(*0*)*=V*_bi_ and *E*_C_(*∞*)*=*0 as the boundary conditions, the energy profile [*E*_C_*(x)*] for Schottky barrier as a function of distance (*x*) from the ferroelectric/semiconductor interface can be deduced as follows^34^


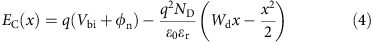


where *φ*_n_ is the difference between the conduction-band minimum (*E*_C_) and the *E*_F_ of NbSTO, defined as *(E*_C_−*E*_F_*)/q*, which is negative (positive) for the degenerated (nondegenerated) NbSTO. *φ*_n_ can be simply estimated using the effective mass of electrons 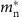 in NbSTO^34^:


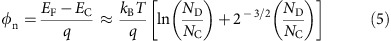



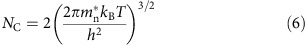


where *N*_C_ is the density-of-state of conduction band of NbSTO and *h* is the Planck constant. The effective Richardson constant of NbSTO in Schottky junctions is, in general, assumed to be 156 A K^−2^ cm^−2^, which corresponds to 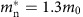 (*m*_0_, electron mass)^38^,^45^.

### Data availability

The data that support the findings of this study are available from the corresponding authors on request.

## Additional information

**How to cite this article:** Xi, Z. *et al*. Giant tunnelling electroresistance in metal/ferroelectric/semiconductor tunnel junctions by engineering the Schottky barrier. *Nat. Commun.* **8**, 15217 doi: 10.1038/ncomms15217 (2017).

**Publisher's note:** Springer Nature remains neutral with regard to jurisdictional claims in published maps and institutional affiliations.

## Supplementary Material

Supplementary Information Supplementary Figures, Supplementary Notes and Supplementary References
