# Supplementary material for: Giant tunnelling electroresistance in metal/ferroelectric/semiconductor tunnel junctions by engineering the Schottky barrier
Source: Nat Commun. 2017 May 17;8:15217. doi: 10.1038/ncomms15217 (PMC5442322; doi:10.1038/ncomms15217)
Supplement: Supplementary Information — Supplementary Figures, Supplementary Notes and Supplementary References [file ncomms15217-s1.pdf]

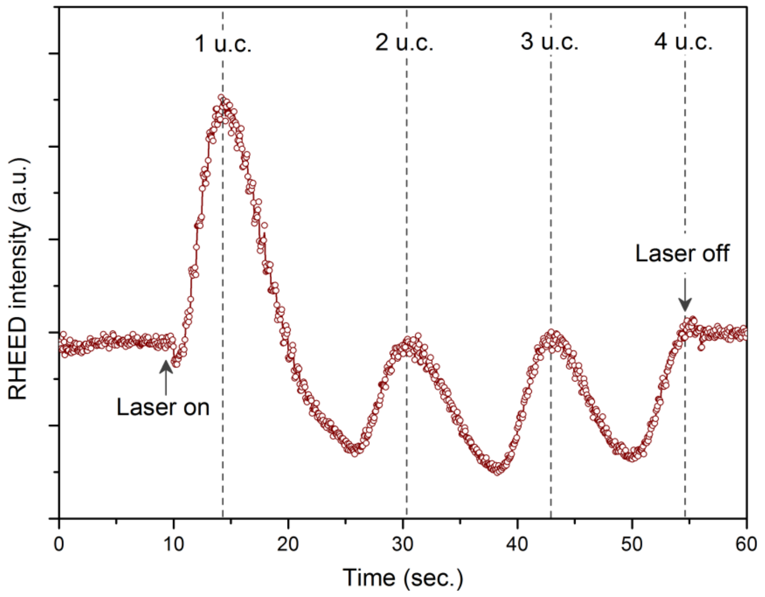

Supplementary Figure 1 | Layer-by-layer growth of the BTO ultrathin film. RHEED intensity oscillations during epitaxial growth of a 4 u.c.-thick BTO on NbSTO substrate.

Supplementary Note 1. As shown in Supplementary Fig. 1, the clear intensity oscillations indicate a layer-by-layer growth of BTO thin film on NbSTO substrate. The thickness of BTO layer can be controlled by counting the number of RHEED intensity oscillations.

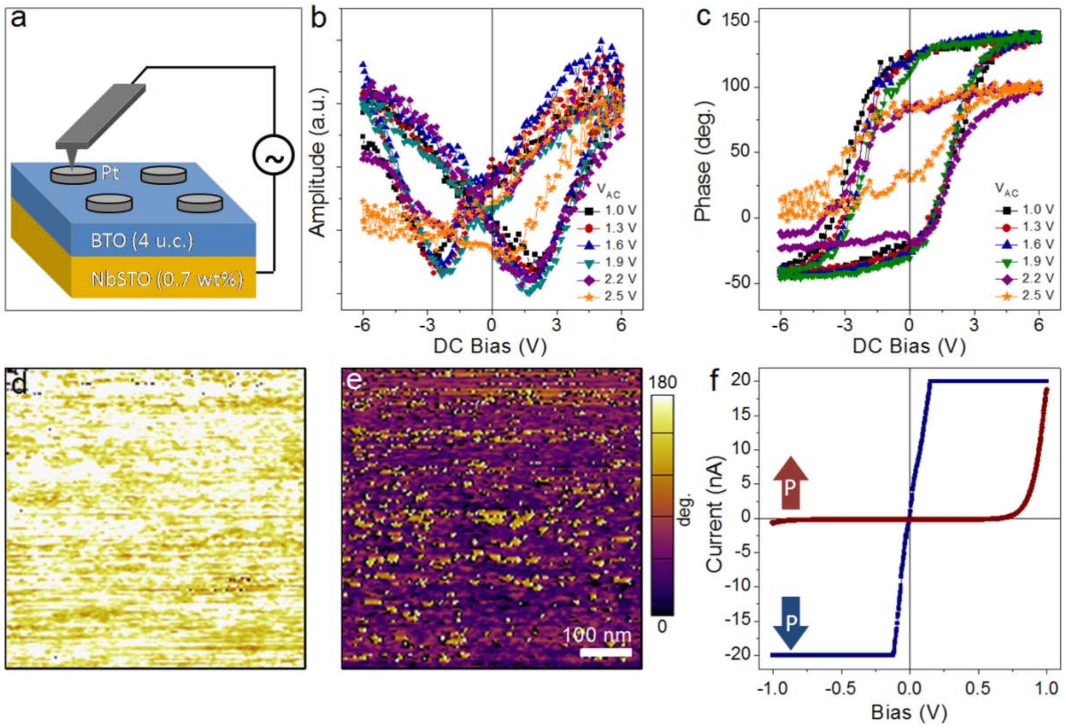

Supplementary Figure 2 | PFM and conductive AFM measurements of a Pt/BTO/NbSTO FTJ. a, Schematic description of the measurements. **b** and **c**, PFM amplitude and phase hysteresis loops measured from a Pt top electrode as a function of V_{AC}. **d** and **e**, PFM phase images recorded on a Pt top electrode after applying a +6.0 and a -6.0 V pulse, respectively. **f**, *I*-*V* curves measured right after the PFM measurements in (**d**) and (**e**) for downward and upward polarized BTO barrier, respectively.

Supplementary Note 2. The V_{AC}-dependent PFM hysteresis loops of a Pt/BTO/NbSTO (Nb: 0.7 wt%) FTJ with a 4 u.c.-thick BTO barrier are shown in Supplementary Fig. 2a-c. Coercive voltages, -2.5 and +1.8 V, can be determined by the minima of the PFM amplitude loop measured with V_{AC}=1.0 V. When V_{AC} is larger than the coercive voltage, both the amplitude and the phase hysteresis loops deform dramatically, in good agreement with that observed from bare BTO/NbSTO heterostructure (Fig. 1b). Supplementary Fig. 2d and 2e show PFM phase images

recorded on the Pt top electrode after applying a +6.0 and a -6.0 V pulse, respectively. In these images, yellow and purple areas with a 180^0 phase contrast represent downward and upward domains. As shown, the effective polarization in the 4 u.c.-thick BTO barrier under the Pt electrode is switched antiparallel. The junction conduction is measured using the same conductive tip with DC bias. As shown in Supplementary Fig. 2f, the device is in the ON state with a larger current when the effective polarization is pointing to the NbSTO substrate. After the polarization is switched, the junction current is suppressed associated with the appearance of a typical rectifying character, indicating that the device is switched to the OFF state with an enhanced Schottky barrier. These results directly correlate the TER characteristics of the Pt/BTO/NbSTO device with the polarization reversal of the BTO barrier. In these measurements, the Pt electrode is only ~5 nm in thickness and a conductive diamond-coated tip is used for both the PFM and the *I-V* measurements. The conductive AFM mode in our facility has a current compliance at 20 nA.

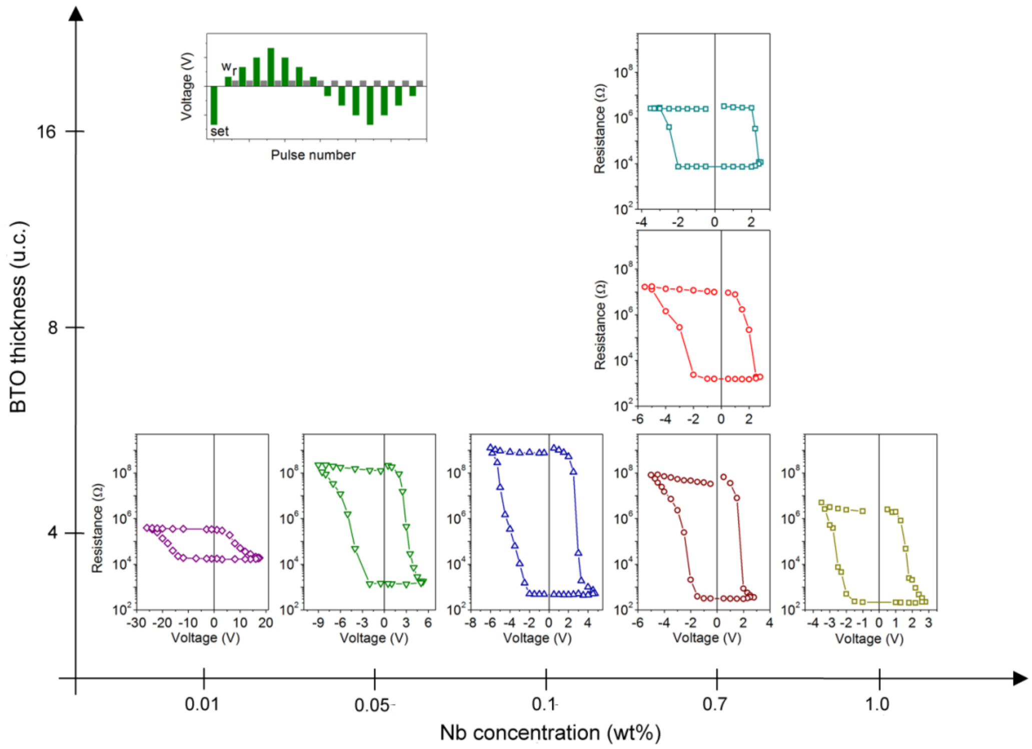

Supplementary Figure 3 | Room-temperature TER of Pt/BTO/NbSTO FTJs. *R*-*V* hysteresis loops as functions of BTO thickness and Nb concentration. The test pulse sequence is shown schematically in the left-top inset, which consists of a set pulse (set), write pulses (w), and readout pulses (r).

Supplementary Note 3. As shown in Supplementary Fig. 3, all Pt/BTO/NbSTO FTJs exhibit typical resistance-voltage (*R*-*V*) hysteresis loops at room temperature following the test pulse sequence shown schematically in the inset. The ON state is achieved by applying positive voltage pulses on the Pt electrode, where the ferroelectric polarization of BTO layer is switched towards the NbSTO electrode. The device is switched to the OFF state by applying negative pulses, in which the BTO polarization is pointing to the Pt electrode and a Schottky barrier appears on the depleted NbSTO surface. The existence of the depletion region also results in an imprint effect. Because a part of write voltage is applied on the depletion region, the

R-*V* loops shift toward negative voltage along the horizontal axis. For example, the coercive voltages of the Pt/BTO/NbSTO (Nb: 0.7 wt%) FTJ with a 4 u.c.-thick BTO barrier are -2.5 and +1.7 V, respectively, in agreement with the PFM hysteresis loops measured using V_{AC}=1.0 V (Supplementary Fig. 2b). The imprint decreases with increasing BTO thickness due to the suppressed depletion region. As shown in Supplementary Fig. 3, the *R*-*V* hysteresis loop becomes symmetric and coercive voltages are -2.4 and +2.2 V, respectively, for the FTJ with a 16 u.c.-thick BTO barrier.

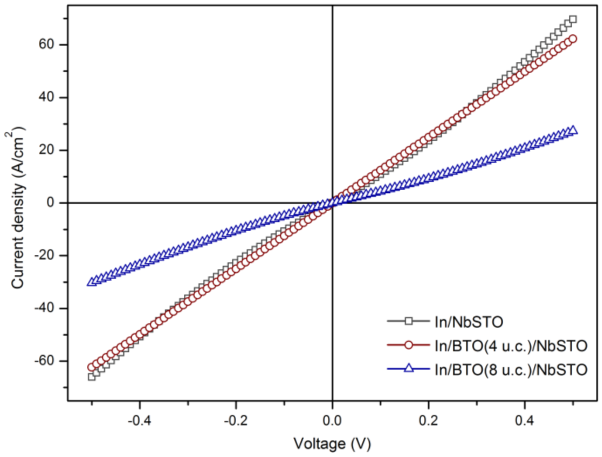

Supplementary Figure 4 | The effect of BTO thickness on electron tunnelling. *J*-*V* curves of In/BTO/NbSTO junctions with a 4 u.c.- and an 8 u.c.-thick BTO barrier, respectively, in comparison with that of an In/NbSTO junction.

Supplementary Note 4. To estimate the resistance of the 4 u.c.-thick BTO on the electron transport, we use indium (In) as the top electrode. Because the work function of In is 4.12 eV, very close to the electron affinity of NbSTO (4.08 eV), the In/NbSTO contact results in a nearly flat band and an Ohmic transport. As shown in Supplementary Fig. 4, the In/BTO/NbSTO junction with a 4 u.c.-thick BTO barrier shows a typical linear *J*-*V* curve, almost identical to that of an In/NbSTO Ohmic contact. This indicates that the 4 u.c.-thick ultrathin BTO barrier shows a negligible resistance to the tunnelling current between In and NbSTO. With this 4 u.c. BTO barrier, if a high work function metal Pt (Φ_{Pt}=5.65 eV) is adopted as the electrode, a Schottky barrier appears and governs the transport through the Pt/BTO/NbSTO FTJ because the BTO barrier itself is negligible to the tunnelling current. This FTJ is actually reduced to a polarization-modulated metal/semiconductor Schottky junction. However, when the BTO thickness increases to 8 u.c., the current density decreases

significantly with an obvious nonlinear character, as observed previously in many FTJs.[1-3] The 8 u.c.-thick BTO shows a prominent resistance to the tunnelling current, in contrast to the 4 u.c.-thick one.

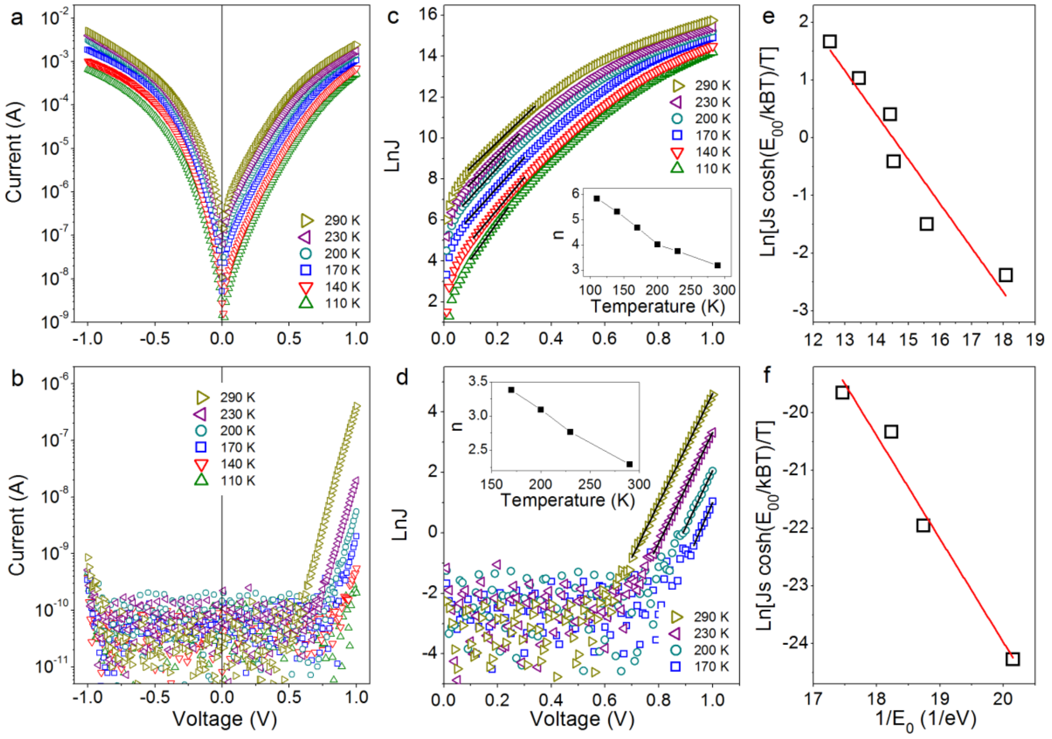

Supplementary Figure 5 | Temperature-dependent transport of a Pt/BTO/NbSTO FTJ. The ON (**a**) and the OFF (**b**) state *I*-*V* curves of a Pt/BTO/NbSTO (Nb: 0.1 wt%) FTJ with a 4 u.c.-thick BTO barrier at various temperature from 290 to 110 K. The forward lnJ_F-V plots for the ON (**c**) and the OFF (**d**) states, where the back solid lines are fits to Supplementary Equation 1. The insets in (**c**) and (**d**) are the ideality factor *n*, extracted from the linear fitting, as a function of temperature. The ln[$J_S$cosh(E_{00}/k_BT)/T]-1/E_0 plots for the ON (**e**) and the OFF (**f**) states, in which the red solid lines are linear fits to Supplementary Equation 2.

Supplementary Note 5. As shown in Supplementary Fig. 5a and 5b, the ON and the OFF state currents of a Pt/BTO/NbSTO (Nb: 0.1 wt%) FTJ with a 4 u.c.-thick BTO barrier decrease monotonically with decreasing temperature from 290 to 110 K because of the suppression of thermal emission. The forward lnJ_F-V plots at various temperatures are shown in Supplementary Fig. 5c and 5d, which can be fitted to Supplementary Equation 1. The ideality factor *n*, extracted from the fitting, is plotted

in the insets as a function of temperature. n increases monotonically, from 3.2 to 5.8 for the ON state and from 2.3 to 3.4 for the OFF state, with decreasing temperature. Similar temperature-dependent behaviors of n have also been observed in NbSTO-based Schottky junctions, indicating the presence of thermally-assisted tunnelling.[4,5] The thermally-assisted tunnelling current can be described by[4-8]

$$J_{\mathrm{F}} = J_{\mathrm{S}}(T)\exp(qV/E_0) \quad (1)$$

$$J_{\mathrm{S}}(T) = \frac{A^{*}T^{2}\pi^{1/2}E_{00}^{1/2}q^{1/2}\left[(V_{\mathrm{bi}}-V)+\phi_{\mathrm{n}}\right]^{1/2}}{k_{\mathrm{B}}T\cosh(E_{00}/k_{\mathrm{B}}T)} \times \exp\left[q\left(\frac{\phi_{\mathrm{n}}}{k_{\mathrm{B}}T} - \frac{V_{\mathrm{bi}}+\phi_{\mathrm{n}}}{E_0}\right)\right] \quad (2)$$

where $E_0 = nk_{\mathrm{B}}T$ and $E_{00} = \frac{qh}{4\pi}\left[\frac{N_{\mathrm{D}}}{m_{\mathrm{n}}^{*}\varepsilon_{\mathrm{r}}(T)\varepsilon_0}\right]^{1/2}$. The relative permittivity $\varepsilon_{\mathrm{r}}(T)$ of NbSTO is a function of temperature, as described by the Barrett's formula,[9]

$$\varepsilon_{\mathrm{r}}(T) = \frac{1635}{\coth\left(\frac{44.1}{T}\right) - 0.937} \quad (3)$$

In this model, the temperature-independent V_{bi} can be extracted from the slope of linear fitting of $\ln[J_{\mathrm{S}}\cosh(E_{00}/k_{\mathrm{B}}T)/T]$ on $1/E_0$, as shown in Supplementary Fig. 5e and 5f. V_{bi} values are 0.8 and 1.7 eV for the ON and the OFF state, respectively, which are comparable to those extracted from the C-V measurement.

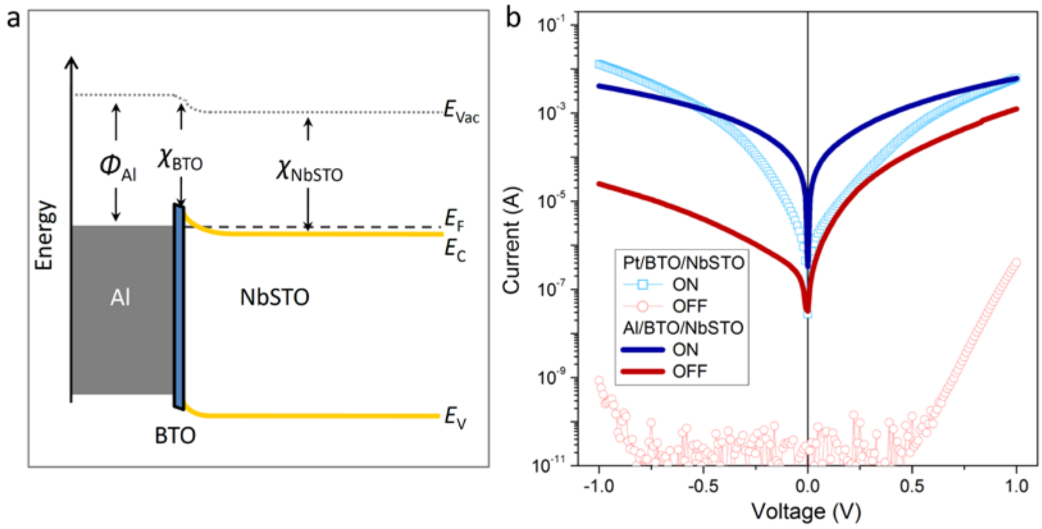

Supplementary Figure 6 | TER character of an Al/BTO/NbSTO FTJ. a, Band alignment of an Al/BTO/NbSTO (Nb: 0.1 wt%) FTJ with a 4 u.c.-thick BTO barrier assumed unpolarized, **b**, *I-V* curves for the ON and the OFF state of the Al/BTO/NbSTO device. The *I-V* curves of a Pt/BTO/NbSTO (Nb: 0.1 wt%) FTJ with a 4 u.c.-thick BTO barrier are also shown in **b** for comparison.

Supplementary Note 6. As depicted in Supplementary Fig. 6a, if the Pt (Φ_{Pt}=5.65 eV) electrode is replaced with a low work function metal, such as Al with Φ_{Al}=4.28 eV, the contact potential decreases and the Schottky barrier is reduced correspondingly. The ON and the OFF state *I-V* curves of an Al/BTO/NbSTO (Nb: 0.1 wt%) FTJ with a 4 u.c.-thick BTO barrier are shown in Supplementary Fig. 6b. It is clear that the TER performance is drastically reduced and the ON/OFF ratio, read at 0.6 V, is only ~10, in contrast to that (~6.0×10^6) in its Pt/BTO/NbSTO counterpart. The small ON/OFF ratio is a result of large OFF state current. Because the Schottky barrier is significantly suppressed due to the low work function of Al, the OFF state transport cannot be shut off even though the BTO polarization is pointing away from NbSTO.

These results evidence the predominant role of the Schottky barrier in TER of MFS type FTJs.

Supplementary References

1. Pantel, D., Goetze, S., Hesse, D., & Alexe, M. Reversible electrical switching of spin polarization in multiferroic tunnel junctions. *Nature Mater.* **11**, 289-293 (2012).
2. Wen, Z., Li, C., Wu, D., Li, A. & Ming, N. Ferroelectric-field-effect-enhanced electroresistance in metal/ferroelectric/semiconductor tunnel junctions. *Nature Mater.* **12**, 617-621 (2013).
3. Hu, W. J., Wang, Z., Yu, W. & Wu, T. Optically controlled electroresistance and electrically controlled photovoltage in ferroelectric tunnel junctions. *Nature Commun.* **7**, 10808 (2016).
4. Cuellar, F. A., Sanchez-Santolino, G., Varela, M., Clement, M., Iborra, E., Sefrioui, Z., Santamaria, J. & Leon, C. Thermally assisted tunneling transport in $La_{0.7}Ca_{0.3}MnO_3/SrTiO_3$:Nb Schottky-like heterojunctions. *Phys. Rev. B* **85**, 245122 (2012).
5. Ruotolo, A., Lam, C. Y., Cheng, W. F., Wong, K. H. & Leung, C. W. High-quality all-oxide Schottky junctions fabricated on heavily doped Nb:$SrTiO_3$ substrates. *Phys. Rev. B* **76**, 075122 (2007).
6. Susaki, T., Kozuka, Y., Tateyama, Y. & Hwang, H. Y. Temperature-dependent polarity reversal in Au/Nb:$SrTiO_3$ Schottky junctions. *Phys. Rev. B* **76**, 155110

(2007).

7. Padovani, F. A. & Stratton, R. Field and thermionic-field emission in Schottky barriers. *Solid-State Electro.* **9**, 695-707 (1966).

8. Sze, S. M. & Ng, K. K. *Physics of Semiconductor Devices* 3rd edn (Wiley, 2007).

9. Barrett, J. H. Dielectric constant in perovskite type crystals. *Phys. Rev.* **86**, 118 (1951).
